# Supplementary material for: Improving Intensive End-of-Life Care for Infants and Children: A Scoping Review of Intervention Elements
Source: Children (Basel). 2025 Nov 3;12(11):1485. doi: 10.3390/children12111485 (PMC12651015; doi:10.3390/children12111485)
Supplement: Supplementary file 1 [file children-12-01485-s001.zip › Supplement_update.pdf]

Supplement

Table of Contents

Search strategy ***2***

Extraction form ***7***

Table of Evidence ***8***

Mixed Methods Appraisal Tool Results ***27***

Search strategy

Ovid MEDLINE(R) ALL <1946 to January 19, 2024>

1	exp adolescent/ or exp child/ or exp infant/
2	(Neonate* or newborn or new born or infant* or child* or adolescen* or baby or babies or toddler* or juvenile* or boy* or girl* or pediatric or paediatric*).mp.
3	1 or 2
4	exp Hospice/ or Palliative Care Nursing/
5	exp Palliative Care/
6	exp Terminal Care/ or exp Patient Comfort/
7	(hospice or palliative or terminal or comfort).mp.
8	(intervention* or education* or train* or knowledge or simulation).ti,ab. and nurs*.mp.
9	4 or 5 or 6 or 7 or 8
10	exp Terminally Ill/
11	exp Death/
12	exp Neoplasms/
13	(tumor* or cancer* or carcino* or tumor* or tumour* or sarcoma* or malignan* or neoplas*).mp.
14	(life threatening or life limiting or terminal or death or dying).mp. or (end adj2 life).ti,ab,kw.
15	exp Chronic Disease/
16	(chronic disease or medical complexity).ti,ab.
17	exp Cardiovascular Diseases/ or cardiovascular disease*.ti,ab.
18	trauma.ti,ab. or exp "Wounds and Injuries"/
19	exp Extracorporeal Membrane Oxygenation/ or (Extracorporeal Membrane Oxygenation or ECMO).mp.
20	10 or 11 or 12 or 13 or 14 or 15 or 17 or 18 or 19
21	(intensive care or ICU or PICU).mp. or exp Intensive Care Units/
22	3 and 9 and 20 and 21

OVID Embase <1974 to 2024 January 19>

1	exp adolescent/ or exp child/ or exp infant/
2	(Neonate* or newborn or new born or infant* or child* or adolescen* or baby or babies or toddler* or juvenile* or boy* or girl* or pediatric or paediatric*).mp.
3	1 or 2
4	exp hospice care/ or exp hospice/ or exp hospice nursing/ or exp hospice patient/
5	exp palliative therapy/
6	exp Terminal Care/ or exp Patient Comfort/
7	(hospice or palliative or terminal or comfort).mp.
8	(intervention* or education* or train* or knowledge or simulation).ti,ab. and nurs*.mp.
9	4 or 5 or 6 or 7 or 8
10	exp Terminally Ill patient/
11	exp Death/
12	exp Neoplasm/
13	(tumor* or cancer* or carcino* or tumor* or tumour* or sarcoma* or malignan* or neoplas*).mp.
14	(life threatening or life limiting or terminal or death or dying).mp. or (end adj2 life).ti,ab,kw.
15	exp Chronic Disease/
16	(chronic disease or medical complexity).ti,ab.
17	exp Cardiovascular Disease/ or cardiovascular disease*.ti,ab.
18	trauma.ti,ab. or exp "Wounds and Injuries"/
19	exp Extracorporeal Oxygenation/ or (Extracorporeal Membrane Oxygenation or ECMO).mp.
20	10 or 11 or 12 or 13 or 14 or 15 or 17 or 18 or 19
21	(intensive care or ICU or PICU).mp. or exp *Intensive Care/
22	3 and 9 and 20 and 21

OVID APAPsycINFO <1806 to January Week 2 2024>

1	(Neonate* or newborn or new born or infant* or child* or adolescen* or baby or babies or toddler* or juvenile* or boy* or girl* or pediatric or paediatric*).mp.
2	exp Hospice/
3	exp Palliative Care/
4	exp Terminally ill patients/ or exp physical Comfort/
5	(hospice or palliative or terminal or comfort).mp.
6	(intervention* or education* or train* or knowledge or simulation).ti,ab. and nurs*.mp.
7	2 or 3 or 4 or 5 or 6
8	exp Terminally Ill patients/
9	exp "Death and Dying"/ or death.ti.
10	exp Neoplasms/
11	(tumor* or cancer* or carcino* or tumor* or tumour* or sarcoma* or malignan* or neoplas*).mp.
12	exp Chronic illness/
13	(chronic disease or medical complexity).ti,ab.
14	exp Cardiovascular disorders/ or cardiovascular disease*.ti,ab.
15	trauma.ti,ab. or exp "Wounds and Injuries"/
16	(Extracorporeal Membrane Oxygenation or ECMO).mp.
17	8 or 9 or 10 or 11 or 12 or 13 or 15 or 16 or "17".mp. [mp=title, abstract, heading word, table of contents, key concepts, original title, tests & measures, mesh word]
18	(intensive care or ICU or PICU).mp. or exp Neonatal Intensive Care/ or exp Intensive Care/
19	or/8-17
20	1 and 7 and 18 and 19

WEB OF SCIENCE Core Collection

TS=(adolescen* or child* or infant* or neonate* or newborn or "new born" or baby or babies or toddler* or juvenile* or boy* or girl* or pediatric or paediatric)
TI=(hospice or palliative or "terminal care" or "patient comfort" or "end of life”)
TS=("terminally ill" or death or neoplasm* or tumor* or cancer* or carcino* or tumor* or tumour* or sarcoma* or malignan* or chronic disease* or "medical complexit*" or cardiovascular disease or trauma or ecmo or "extracorporeal membrane oxygenation”)
#1 AND #2 AND #3

COCHRANE Central

(adolescen* or child* or infant* or neonate* or newborn or "new born" or baby or babies or toddler* or juvenile* or boy* or girl* or pediatric or paediatric)
TS=("terminally ill" or death or neoplasm* or tumor* or cancer* or carcino* or tumor* or tumour* or sarcoma* or malignan* or chronic disease* or "medical complexit*" or cardiovascular disease or trauma or ecmo or "extracorporeal membrane oxygenation”)
terminally near/2 ill or death or neoplasm* or tumor* or cancer* or carcino* or tumor* or tumour* or sarcoma* or malignan* or chronic near/2 disease* or medical next/2 complexit* or cardiovascular near/2 disease or trauma or ecmo or extracorporeal near/4 oxygenation
#1 AND #2 AND #3 [In TRIALS]

CINAHL

S23	S3 AND S8 AND S19 AND S22	
S22	S20 OR S21	
S21	"intensive care" OR "PICU" OR "NICU" OR "ICU"	
S20	(MH "Intensive Care, Neonatal+") OR (MH "Intensive Care Units+") OR (MH "Intensive Care Units, Neonatal") OR (MH "Intensive Care Units, Pediatric+")	
S19	S9 OR S10 OR S11 OR S12 OR S13 OR S14 OR S15 OR S16 OR S17 OR S18	
S18	(MH "Extracorporeal Membrane Oxygenation") OR "Extracorporeal Membrane Oxygenation" OR "ECMO"	
S17	(MH "Trauma+") OR "trauma"	
S16	(MH "Cardiovascular Diseases+") OR "cardiovascular disease"	
S15	chronic disease or medical complexity	
S14	(MH "Chronic Disease+")	
S13	life threatening or life limiting or terminal or death or dying OR (end N3 life)	
S12	tumor* or cancer* or carcino* or tumor* or tumour* or sarcoma* or malignan* or neoplas*	
S11	(MH "Neoplasms+")	
S10	(MH "Death+")	
S9	(MH "Terminally Ill Patients+")	
S8	S4 OR S5 OR S6 OR S7	
S7	hospice or palliative or terminal or comfort	
S6	(MH "Terminal Care+") OR (MH "Terminally Ill Patients+")	
S5	(MH "Palliative Care") OR (MH "Palliative Care Nursing") OR (MH "Palliative Care Nurses") OR (MH "Palliative Medicine")	
S4	(MH "Hospice Nurses") OR (MH "Hospice Patients") OR (MH "Hospice Nursing") OR (MH "Hospice Care") OR (MH "Hospices")	
S3	S1 OR S2	
S2	Neonate* or newborn or new born or infant* or child* or adolescen* or baby or babies or toddler* or juvenile* or boy* or girl* or pediatric or paediatric*	
S1	(MH "Child+") OR (MH "Adolescence+") OR (MH "Infant+")	

Extraction form

Data Extraction Form

Please complete the survey below.

Thank you!

Title	__________________ (Title of study)
Authors:	__________________ (last name, first initial of first three authors)
Publication year	__________________ (copy from methods or publication year)
Journal.	__________________ (full name of journal)
Volume	__________________
Issue	__________________
Pages	__________________
DOI	__________________
Study design	◯ qualitative<br>◯ quantitative RCT<br>◯ quantitative non-randomized<br>◯ quantitative descriptive<br>◯ mixed methods<br>◯ other (please describe)<br>(i.e. pre-post, quasi experimental, RCT, feasibility, etc.)
Other study design:	__________________ (i.e. pre-post, quasi experimental, RCT, feasibility, etc.)
Describe intervention (development, content, etc.)	__________________ (copy and paste from article)
Intervention modality	__________________ (i.e. type of intervention: education, symptom management, communication, etc.)

Intervention dose/duration/frequency	__________________________________<br>(if reported, how much, how long, and how often is the intervention delivered?)
Targeted PRIMARY outcome	__________________________________<br>(What outcomes are assessed? What measures or qualitative questions (e.g. experience of early bereavement, attitudes and beliefs about palliative care)?)
Additional outcomes	__________________________________<br>(What outcomes are assessed? What measures or qualitative questions (e.g. experience of early bereavement, attitudes and beliefs about palliative care)?)
Effect on PRIMARY outcome	__________________________________<br>(Positive effect, negative effect, other (describe: unclear, qualitative, etc.))
Other effects	__________________________________<br>(Positive effect, negative effect, other (describe: unclear, qualitative, etc.))
Delivery	__________________________________<br>(how is it delivered (EHR, modules, learning, etc.))
Fidelity	__________________________________<br>(What does the article report about fidelity (i.e. extent to which intervention was delivered as intended)?)
Palliative care domain	☐ None<br>☐ Physical<br>☐ Emotional/psychological<br>☐ Spiritual<br>☐ Social
Interventionist role	__________________________________<br>(Who delivers the intervention? (PICU physician, Palliative care physician, social worker, nurse, etc.))
Recipient role	__________________________________<br>(who receives the intervention? (Parent, child, nurse, physician, etc.))

Sample size: include ENTIRE sample and % in ICU if needed	__________________ (number of participants in ENTIRE study)
Child's diagnoses	__________________ (diagnoses or reported diseases)
Who makes up the study sample?	☐ Children<br>☐ Parents/families<br>☐ Clinicians
Child race (N and % of groups)	__________________ (Number and frequencies of child race if reported)
Child ethnicity (N and % of groups)	__________________ (frequencies of child ethnicity if reported)
Mean (or median) and range of child ages	__________________ (child age in years if reported)
Child gender (N and % of groups)	__________________ (frequencies of child gender if reported)
Parent race (N and % of groups)	__________________ (frequencies of parent race if reported)
Parent ethnicity (N and % of groups)	__________________ (frequencies of parent ethnicity if reported)
Parent gender (N and % of groups)	__________________ (frequencies of parent gender if reported)
Socioeconomic status: include percentages of socioeconomic variables (education, income, etc.) if reported	__________________
Primary language spoken by family	__________________ (primary language spoken by family if reported, or language-based exclusion criteria)
Clinician race (N and % of groups)	__________________ (frequencies of clinician race if reported)
Clinician ethnicity (N and % of groups)	__________________ (frequencies of clinician ethnicity if reported)

Clinician gender (N and % of groups)	__________________________________ (frequencies of clinician gender if reported)
Clinician experience (N and % of groups)	__________________________________ (any information about clinician experience)
Clinical disciplines (N and % of groups)	__________________________________ (Any reported information about disciplines (i.e. frequencies of RNs, NPs, MDs, RTs, etc))
Subspecialty palliative care	__________________________________ (Any reported information about subspecialty palliative care)
Unit type	☐ general PICU<br>☐ cardiac PICU<br>☐ other speciality PICU (heme-onc, neuro, trauma, etc.)<br>☐ NICU<br>☐ other<br>(general PICU, PCICU, surgical ICU, neuro ICU, NICU)
Unit level implementation context: facilitators, barriers	____ (Any reported info about facilitators and barriers to implementation at the unit level (i.e. challenges with teamwork, staffing, ease of use, unit policies, unit physical environment, etc.))
Implementation outcomes: adoption	__________________________________ (any reported info about uptake of intervention)
Implementation outcomes: sustainment	__________________________________ (any reported info about continuation of intervention after study)
Hospital characteristics	____ (info about hospital: academic, community, childrens, for profit, number of picu beds, etc.)
Geographic hospital location	__________________________________ (state or region of hospital)
Hospital implementation context: facilitators and barriers	____ (any information about hospital level implementation challenges or supports)

Are there clear research questions?

◯ Yes
◯ No
◯ can't tell

Do the collected data allow to address the research questions?

◯ Yes
◯ No
◯ Can't tell

MMAT - Qualitative

	yes	no	can't tell
Is the qualitative approach appropriate to answer the research question?	◯	◯	◯
Are the qualitative data collection methods adequate to address the research question?	◯	◯	◯
Are the findings adequately derived from the data?	◯	◯	◯
Is the interpretation of results sufficiently substantiated by data?	◯	◯	◯
Is there coherence between qualitative data sources, collection, analysis and interpretation?	◯	◯	◯

Explain any "no" or "can't tell" answers: ______________________________

MMAT - RCT

	yes	no	can't tell
Is randomization appropriately performed?	◯	◯	◯
Are the groups comparable at baseline?	◯	◯	◯
Are there complete outcome data?	◯	◯	◯
Are outcome assessors blinded to the intervention provided?	◯	◯	◯
Did the participants adhere to the assigned intervention?	◯	◯	◯

Explain any "no" or "can't tell" answers: ______________________________

MMAT - Quant (non-RCT)

	yes	no	can't tell
Are the participants representative of the target population?	◯	◯	◯
Are measurements appropriate regarding both the outcome and intervention (or exposure)?	◯	◯	◯
Are there complete outcome data?	◯	◯	◯
Are the confounders accounted for in the design and analysis?	◯	◯	◯
During the study period, is the intervention administered (or exposure occurred) as intended?	◯	◯	◯

Explain any "no" or "can't tell" answers: __________________________________

MMAT - Quantitative Descriptive

	yes	no	can't tell
Is the sampling strategy relevant to address the research question?	◯	◯	◯
Is the sample representative of the target population?	◯	◯	◯
Are the measurements appropriate?	◯	◯	◯
Is the risk of nonresponse bias low?	◯	◯	◯
Is the statistical analysis appropriate to answer the research question?	◯	◯	◯

Explain any "no" or "can't tell" answers: __________________________________

MMAT- Mixed Methods

	yes	no	can't tell
Is there an adequate rationale for using a mixed methods design to address the research question?	◯	◯	◯

Are the different components of the study effectively integrated to answer the research question?	◯	◯	◯
Are the outputs of the integration of qualitative and quantitative components adequately interpreted?	◯	◯	◯
Are divergences and inconsistencies between quantitative and qualitative results adequately addressed?	◯	◯	◯
Do the different components of the study adhere to the quality criteria of each tradition of the methods involved?	◯	◯	◯

Explain any "no" or "can't tell" answers: __________________________________

Reviewed

◯ EB
◯ IE
◯ NO
◯ SW

Additional Review Required?

◯ Yes
◯ No

Table of Evidence

Title	Authors, Year	Country[a]	Design	Intervention description	Primary outcome	Sample	Unit
"Most Prized Possessions": Photography as Living Relationships Within the EOL Care of Newborns	(Martel & Ives-Baine, 2014)	Canada	qualitative	"parents who had experienced the death of their newborn ...participated in EOL photography, having photos taken by nurses, by themselves and/or professional photographers."	Experience of EOL photography	10 parents	NICU
Meaning making during parent-physician bereavement meetings after a child's death	(Meert et al., 2015)	USA	qualitative	"bereavement meeting to provide parents with an opportunity to gain information about their child's illness and hospital course, ask questions, and provide feedback on their hospital experiences."	parental meaning making processes during bereavement meeting	53 parents	PICU
Implementing Palliative care, based on family-centered care, in a highly complex neonatal unit	(Morillo Palomo et al., 2023)	Spain	quantitative non-randomized	Protocol including criteria for recommending transition of care, support throughout the care process (clinical controls and care, monitoring, changes in treatment, analgesia and sedation, environment and attention to the family), post-mortem care, and family follow-up.	Diagnoses, cause of death, medical examination, organ donation, transition of care, sedation, place of death, family rituals, palliative care referrals	344 patients	NICU

Development of a Neonatal End-of-Life Care Education Program for NICU Nurses in Japan	(Murakami et al., 2015)	Japan	quantitative non-randomized	educational program consisting of 6 modules (intro, decision making, EOL care, bereavement care, communication, support for nurses)	knowledge test about neonatal EOL care	30 nurses	NICU
The Use of Simulation to Improve Resident Communication and Personal Experience at End-of-Life Care	(Nellis et al., 2017)	USA	quantitative non-randomized	Simulation of an unsuccessful resuscitation featuring trained actors in the roles of a standardized parent and nurse. Primary learning objective was "to demonstrate both empathy and strong interpersonal skills in communicating with a parent of a child at the EOL	Self perceived communication competency	31 residents	PICU, NICU
A comprehensive pediatric bereavement program: The patterns of your life	(Nesbit et al., 1997)	USA	Other: quality improvement	Comprehensive checklist of nursing tasks including emotional support, symptom management, communication, care coordination), educational resources, and family follow-up for 1 year.	feedback from families	63 families	PICU

Mindful Movement: Tai Chi, Gentle Yoga, and Qi Gong for Hospitalized Pediatric Palliative Care Patients and Family Members	(Parry et al., 2018)	USA	quantitative non-randomized	Gentle yoga, tai chi, and qi gong at the bedside of pediatric palliative care patients with inclusion of child and family	overall wellness level	17 family caregivers 7 pediatric palliative care patients	PICU and other
Educational interventions in end-of-life care: Part I: An educational intervention responding to the moral distress of NICU nurses provided by an ethics consultation team	(Rogers et al., 2008)	USA	quantitative non-randomized	an educational intervention focused on the clinical, ethical, and legal issues surrounding neonatal care. The content of the program was based on the ELNEC/IPPC curricula. Topics included pain management; symptom management; ethical/legal issues; communication/ culture; spiritual/anxiety issues at end-of-life; and prevention of compassion fatigue.	the level of comfort of NICU nurses caring for dying infants	82 nurses	NICU
Interdisciplinary interventions to improve pediatric palliative care and reduce health care professional suffering	(Rushton et al., 2006)	USA	Other: quality improvement	Four elements: The first intervention established a compassionate care network to integrate palliative and end-of-life care information and expertise across all units in the Children's Center. The remaining three interventions were palliative care rounds, patient care conferences, and	Feasibility and utilization	950 participants (including review of patient charts, clinician feedback)	PICU, NICU, and other

				bereavement debriefing sessions.			
End-of-life care in a regional level IV neonatal intensive care unit after implementation of a palliative care initiative	(Samsel & Lechner, 2015)	USA	quantitative descriptive	Interprofessional (MD, RN, APRN, RT, OT, pharm, etc.) committee developed three main foci for the NICU palliative care intervention: provider education, formal practice guideline development and communication skills training	redirection of care and palliative medication usage	106 neonates	NICU
Pediatric End-of-Life Care Skills Workshop: A Novel, Deliberate Practice Approach	(Scheurer et al., 2023)	USA	quantitative non-randomized	Simulation workshop including a full-group pre-brief; two immersive simulations with standardized patients and short, structured debriefings with formative feedback; a skills station; and a full-group debrief. Technical skills included symptom management, medication dosing and escalation, extubation, communication, anticipatory guidance	pediatric end-of-life care skills (PECS) self-efficacy	34 fellows, advanced practice providers, 12 neonatal, 6 pediatric critical care	PICU, NICU

Analgesia and Sedation at Terminal Extubation: A Secondary Analysis from Death One Hour after Terminal Extubation Study Data*	(Tripathi et al., 2023)	USA	quantitative descriptive	pharmacologic symptom management following terminal extubation ("TE was defined as the discontinuation of invasive mechanical ventilation with the expectation that death would occur without intent for reintubation or cardiopulmonary resuscitation.")	time to death after TE in minutes (excluded if greater than 1 hour post TE)	680 patients	PICU, PCICU
Palliative care education in neonatal units: impact on knowledge and attitudes	(Twamley et al., 2013)	United Kingdom	quantitative non-randomized	The educational program included sessions about principles of palliative care, available services/resources, useful documents	knowledge of palliative care services and resources, attitudes to palliative care, confidence in skills related to palliative care,	264 total, 193 (73%) NICU clinicians	NICU
Bringing Home to the Hospital: Development of the Reflection Room and Provider Perspectives	(Vesely et al., 2017	USA	quantitative descriptive	Alternative homelike location, called the reflection room for EOL care to provide an alternative location for EOL care for the last 8-12 hours of life and for up to 24 hours of postmortem (PM) care. The palliative care team was available for additional support and anticipatory guidance for providers, the child, and their family.	describe patients who used the RR and feedback from providers who cared for patients in the RR	116 patients/families (90 from PICU/NICU); 201 clinicians (27% PICU and 37% NICU)	PICU, NICU

End of Life Simulation in a Pediatric Cardiac Intensive Care Unit	(Williams et al., 2021)	USA	Other: quality improvement	EOL simulation team was created to improve EOL education for nurses undergoing orientation to the CICU. Simulation focused on communication with family during the resuscitation event, while the family was making the decision to withdraw care, and during EOL and post mortem care.	experience with EOL care and their opinions about EOL education in the unit.	69 nurses	PCICU
Chameleon project: a children's end-of-life care quality improvement project	(Wolff et al., 2021)	United Kingdom	Other: quality improvement	Added specialist Pediatricians and Nurse Specialist, education and training programmes, and clinical champions and shared resources including PPC handbook, online guidelines. Brochures about how to access PPC supplied.	12 month evaluation: place of death, admissions, bed days, admission costs	101 children, 24 staff, 4 parents	PICU, NICU
Impact of a palliative care program on end-of-life care in a neonatal intensive care unit	(Younge et al., 2015)	USA	quantitative descriptive	The program includes a palliative care protocol, an electronic order set that can be individualized, a nursing plan of care, staff education and medication guidelines for palliative and end-of-life care including use of sedatives, analgesics and muscle relaxants	family meetings, morphine dosage, use of benzodiazepines, use of neuromuscular blockers, do-not-resuscitate orders in chart at the time of death) and withdrawal of life support	150 patients	NICU

Testing a Family Supportive End of Life Care Intervention in a Chinese NICU: A Quasi-experimental Study With a Non-randomized Controlled Trial Design	(Zhang et al., 2022)	China	quantitative non-randomized	a separate single bed room with enough space for parents to stay comfortably on a sofa. parents were encouraged to be involved in infants basic physical care. nurses helped create commemorative items. trained psychologist and neonatologist had daily interviews with parents to identify needs and provide emotional support.	depression and satisfaction as reported by parents at one week after infant's death.	45 infants and 90 parents	NICU
Impact of educational programs on nurses' knowledge and attitude toward pediatric palliative care	(Abuhammad & Almasri, 2022)	Jordan	quantitative non-randomized	educational program with eight sections: introduction, pain assessment and management, communication with children and emotional issues, child development and play in PPC, grief and bereavement, end-of-life care, perinatal palliative care, and symptoms other than pain in PPC.	nurses knowledge and attitudes towards pediatric palliative care	120 total, 23 (19%) NICU and 22 (18%) PICU nurses	PICU, NICU
Improving Neonatal Intensive Care Unit Providers' Perceptions of Palliative Care through a Weekly Case-Based Discussion	(Allen et al., 2021)	USA	quantitative non-randomized	Case discussion including NICU update including the following: a brief overview of the medical course, code status, anticipated need for surgical intervention, and likelihood of survival to discharge. Following the NICU update, a structured discussion using a standardized template was facilitated by the study investigators and guided by the palliative care domains of pain	NICU staff perspectives (current practice, personal beliefs, delivery of PC) on palliative care.	31 clinicians	NICU

				and symptom management, goals of care, spiritual support, and psychosocial aspects including strengths of and challenges for the patient's family.			
Multidisciplinary Simulation in Pediatric Critical Care: The Death of a Child	(Youngblood et al., 2012)	USA	Other: general implementation evaluation	pediatric death and dying course involved 3 separate scenarios involving the death of a child. Each multidisciplinary team had to share with the family the death of the child, or in the case of the PICU scenario, that the child was dying and that nothing more could be done. After each simulation was completed, teams participated in a 30minute debriefing session.	experience and feedback	not reported	PICU

Legacy Building in Pediatric End-of-Life Care through Innovative Use of a Digital Stethoscope	(Andrews et al., 2020)	USA	Other: implementation evaluation	Digital keepsake created for surviving family members by recording the heart sounds of dying children. Music Therapy was consulted to record the child's heartbeat and edit ambient noise to isolate the heartbeat. The heartbeat was overlaid to a song or voice recording of the family or child or kept as a stand-alone file. Child life specialists and art therapists also created an artistic embellishment of the phonocardiogram	parents general experience with program	12 parents from 11 families	PICU
A Curriculum to Improve Residents' End-of-Life Communication and Pain Management Skills During Pediatrics Intensive Care Rotation: Pilot Study	(Asuncion et al., 2013)	USA	quantitative non-randomized	weekly education curriculum of short lectures, small group discussion, role play, and reflection. goals were to improve residents' confidence and ability to (1) conduct EOL conversations with parents, (2) manage withdrawal of care, and (3) assess and manage pain. Topics included effective communication personal coping, and pain management	self-assessment confidence and competency	17 residents (all rotated through PICU)	PICU
The wrap-up: A unique forum to support pediatric residents when faced with the death of a child	(Bateman et al., 2012)	USA	quantitative descriptive	a unique multidisciplinary guided debriefing within 48 hours of each pediatric intensive care unit death, multidisciplinary (all care providers were invited)	general evaluation of program (Likert style questions)	27 residents	PICU

The Use of Dexmedetomidine in Pediatric Palliative Care: A Preliminary Study	(Burns et al., 2017)	USA	quantitative non-randomized	administration of dexmedetomidine during EOL care	average daily pain score	9 (7, 78% in ICU) patients	PICU, PCICU
Utility of morbidity and mortality conference in end-of-life education in the neonatal intensive care unit	(Carter & Guthrie, 2007)	USA	quantitative descriptive	integration of EOL education and reflection into routine morbidity and mortality conference (though does not describe anything beyond reflection)	documentation of interdisciplinary palliative and EOL care	26 cases (26 patients)	NICU
Using Quality Improvement Science to Create a Navigator in the Electronic Health Record for the Consolidation of Patient Information Surrounding Pediatric End-of-Life Care	(Casas et al., 2021)	USA	Other: quality improvement	EHR navigator to consolidate post mortem documentation and psychosocial care developed through PDSA cycles including focus groups and iterative intervention development	documentation surrounding code status changes.	only aggregate data (percentages) reported	PICU, NICU, PCICU

Copying medical summaries on deceased infants to bereaved parents	(Clarke & Booth, 2011)	United Kingdom	quantitative descriptive	parents received an identical copy of their infant's medical summary as sent to their doctor. Each summary contained a full, detailed medical account of the perinatal history, clinical course, neonatal problems, and circumstances of death, and also verbatim details of the official death certificate.	parent opinion about receiving letter (Likert scale)	13 parents, 32 neonatologists, 21 primary care providers	NICU
Are the GFRUP's recommendations for withholding or withdrawing treatments in critically ill children applicable? Results of a two-year survey	(Cremer et al., 2007)	France	quantitative descriptive	working group of PICU nurses and physicians, parents, palliative care specialists, philosophers, and persons that had conducted ethics research created a guideline for decisions to withdraw life sustaining treatments	number children who underwent decision making procedure, timing of decision making procedure, disposition after decision making procedure	55 children	PICU
The Mother Baby Comfort Care Pathway: The Development of a Rooming-In-Based Perinatal Palliative Care Program	(Czynski et al., 2022)	USA	quantitative descriptive	Special needs care plan in electronic medical record and workshops (on perinatal palliative and end-of-life care for staff and comfort care pathway perinatal palliative care guidelines, posted to hospital wide intranet with access for all staff members	satisfaction with comfort care pathway	2 families, 7 nurses	NICU

Feasibility of a Comfort Care Protocol Using Oral Transmucosal Medication Delivery in a Palliative Neonatal Population	(Drolet et al., 2016)	Canada	quantitative descriptive	standardized comfort care protocol (standard doses of oral or transmucosal medications, standard assessment of pain, respiratory distress, anxiety)	feasibility (goal >80% completion, 75% followed per protocol)	12 neonates, 27 nurses	NICU
Neonatal loss in the intensive care nursery. Effects of maternal grieving and a program for intervention	(Harmon et al., 1984)	USA	qualitative	"family room" for more privacy and homelike environment for terminally ill infants, increased frequency of family communication/conferences, hospice course for staff in NICU, monthly staff reflection/debrief meetings, systematic follow up with families	qualitative questions about impact of loss on individual mother, relationship with spouse	19 mothers pre, 19 mothers post	NICU
A Structured End-of-Life Curriculum for Neonatal-Perinatal Postdoctoral Fellows	(Harris et al., 2015)	USA	quantitative non-randomized	Didactic sessions entitled "Improving End-of-Life Communication in the NICU," "Addressing Pain Management and Personal/Professional Challenges," and "Core Concepts in Neonatal Ethics" and 1 hour per week educational multidisciplinary palliative care bedside rounds Laminated cards detailing the primary aspects of communication, documentation, and medical	usefulness of the sessions, knowledge regarding patient qualification for comfort care and withdrawal of support, knowledge of appropriate end-of-life medical management, comfort levels addressing the	17 fellows	NICU

				care at the end of life were also made available for the fellows.	family, patient pain assessment		
Implementing a Program to Improve Pediatric and Pediatric ICU Nurses' Knowledge of and Attitudes Toward Palliative Care	(Haut et al., 2012)	USA	quantitative non-randomized	A modified, online version of the pediatric (ELNEC-PPC. The modified version used content from nine of the 10 modules, which included an introduction; pediatric considerations in palliative care; communication; ethical, legal, and cultural considerations; pain and symptom management; and grief and loss issues	knowledge and attitudes of pediatric palliative and EOL issues	total n=25, PICU n=21 (85.7%)	PICU

Providing Support for Neonatal Intensive Care Unit Health Care Professionals: A Bereavement Debriefing Program	(Hawes et al., 2022)	USA	quantitative non-randomized	Debriefing sessions within 72 hours after the death facilitated by a chaplain or psychiatric clinical nurse specialist. The main topics in the debriefing session included: basic facts, case review, emotional components of the case, grief responses, wellness strategies for navigating grief, reflection, and conclusion	attendance, stress levels, satisfaction about EOL care	pre: n=11; post: n=39 NICU staff	NICU
An intensive, simulation-based communication course for pediatric critical care medicine fellows	(Johnson et al., 2017)	USA	quantitative descriptive	Faculty led short didactic sessions, faculty demonstration of core communication skills, and simulation with actors specifically trained for roles as patient parents focused on progression from initial family meeting to discussion or death/limitation of LST	evaluate the course's effect on confidence in their communication skills	38 fellows	PICU
Development and Evaluation of a Self-Reflection Program for Intensive Care Unit Nurses Who Have Experienced the Death of Pediatric Patients	(Kang & Bang, 2017)	South Korea	mixed methods	Education program including six topics: What is my emotional pattern, Sharing experiences of caring for dying children, sharing my breakup experience, farewell well: finding your own bereavement consciousness, understanding parents' minds, establishing a self-care strategy	personal growth score	38 nurses, (experimental group=15, control group=23)	PICU, NICU

A Network Approach to Neonatal Palliative Care Education Impact on Knowledge, Efficacy, and Clinical Practice	(Knighting et al., 2019)	United Kingdom	mixed methods	full day interactive workshop developed by neonatal NP, pediatric medical and palliative care consultants, and a nurse	self-efficacy and thanatophobia	73 (61 from neonatal unit/NICU)	NICU
Changes in the End-of-Life Process in Patients with Life-Limiting Diseases through the Intervention of the Pediatric Palliative Care Team	(Kwon & Kim, 2023)	South Korea	quantitative non-randomized	Piloted a palliative care project including services such as physical and psychosocial support, assistance in the decision-making process, end-of-life care, and support for bereaved families.	changes in EOL care processes (advanced care planning, decisions about withholding or withdrawing LST, intubation, CPR, place of death)	12 families, 48 children (72% died in ICU in period 1, ~25-30% in period 2)	PICU
Skin-to-skin care for dying preterm newborns and their parents - A phenomenological study from the perspective of NICU nurses	Kymre & Bondas, 2013)	Sweden, Denmark, Norway	qualitative	skin to skin care during dying process for neonates	how nurses enact skin to skin care for dying preterm newborns	18 NICU nurses	NICU

An exploration of Neonatal Intensive Care Unit (NICU) staff experiences of attending pre-brief and debrief groups surrounding a patient's death or redirection of care	(Woolgar & Archibald, 2021)	United Kingdom	mixed methods	NICU staff support groups including clinical psychologists facilitated pre-brief and debrief protocols. Suppot groups incorporated essential elements of trauma interventions; promoting a sense of 1) safety, 2) calm, 3) self-efficacy, 4) connectedness and 5) hope.	'staff experience survey'. staff experiences of the combined 'staff support groups'	33 NICU staff	NICU
Effect of Educational Program on Nurses' Performance Regarding Neonatal Palliative Care	(Sabaq & Khalaf, 2016)	Egypt	quantitative non-randomized	The educational program consisting of two parts, theoretical (definition of palliative care, benefits, principles, reasons, philosophy, and role of nurse regarding palliative care) and the practical (the nursing role related to control of pain, comfort measures, symptom management, ventilation therapy, oxygen therapy, nutrition, and support of family).	nurses' knowledge, practice and attitude regarding neonatal palliative care	142 nurses	NICU

Palliative Care for Newborns in India: Patterns of Care in a Neonatal Palliative Care Program at a Tertiary Government Children's Hospital	(Ishak Tayoob et al., 2024)		Other: multi-method (no integration of quantitative and qualitative)	The NPC program provided inpatient consultations for any child admitted to the NICU. The care provided included standardized assessment of pain and other symptoms. Common palliative nursing care issues which the team addressed included skin care, feeding and general newborn care. The team also focused on ensure that families received adequate communication about the child's condition and prognosis, in coordination with the neonatology team. Psychosocial support, counseling, and telephone support after discharge were also provided. Bereavement support was mainly provided by telephone, with in-person visits when feasible.	Experiences of neonatal palliative care team implementation, number of referrals and timing of death	110 neonates: 89 (90.9%) who died, unclear N for clinicians	NICU

Grieving Children' Death in an Intensive Care Unit: Implementation of a Standardized Process	(Delgado - Corcoran et al., 2023)		quantitative descriptive	Psychologist moderated staff support sessions began with a moment of silence, honoring the children who had died since the previous session. The remainder of the session was an open discussion that began with a ''check-in'' with each participant. These check-ins informed subsequent discussion topics and bereavement work.	single-item burnout measure	103 PICU and PCICU staff	PICU, PCICU

Note: where the study was conducted or first author institution country if unable to determine from article text

References:

Abuhammad, S., & Almasri, R. (2022). Impact of educational programs on nurses' knowledge and attitude toward pediatric palliative care. *Palliative & Supportive Care*, *20*(3), 397–406. https://doi.org/10.1017/s1478951521001139

Allen, J. D., Shukla, R., Baker, R., Slaven, J. E., & Moody, K. (2021). Improving Neonatal Intensive Care Unit Providers' Perceptions of Palliative Care through a Weekly Case-Based Discussion. *Palliative Medicine Reports*, *2*(1), 93–100. https://doi.org/10.1089/pmr.2020.0121

Amber, Q., Lynn Zinkan, J., Tofil, N. M., & White, M. L. (2012). Multidisciplinary Simulation in Pediatric Critical Care: The Death of a Child. *Critical Care Nurse*, *32*(3), 55–61. https://doi.org/10.4037/ccn2012499

Andrews, E., Hayes, A., Cerulli, L., Miller, E. G., & Slamon, N. (2020). Legacy Building in Pediatric End-of-Life Care through Innovative Use of a Digital Stethoscope. *Palliative Medicine Reports*, *1*(1), 149–155. https://doi.org/10.1089/pmr.2020.0028

Asuncion, A. M., Cagande, C., Schlagle, S., McCarty, B., Hunter, K., Milcarek, B., Staman, G., Da Silva, S., Fisher, D., & Graessle, W. (2013). A Curriculum to Improve Residents' End-of-Life Communication and Pain Management Skills During Pediatrics Intensive Care Rotation: Pilot Study. *Journal of Graduate Medical Education*, *5*(3), 510–513. https://doi.org/10.4300/JGME-D-12-00148.1

Bateman, S. T., Dixon, R., & Trozzi, M. (2012). The wrap-up: A unique forum to support pediatric residents when faced with the death of a child. *Journal of Palliative Medicine*, *15*(12), 1329–1334. https://doi.org/10.1089/jpm.2012.0253

Burns, J., Jackson, K., Sheehy, K. A., Finkel, J. C., & Quezado, Z. M. (2017). The Use of Dexmedetomidine in Pediatric Palliative Care: A Preliminary Study. *Journal of Palliative Medicine*, *20*(7), 779–783. https://doi.org/10.1089/jpm.2016.0419

Carter, B. S., & Guthrie, S. O. (2007). Utility of morbidity and mortality conference in end-of-life education in the neonatal intensive care unit. *Journal of Palliative Medicine*, *10*(2), 375–380. https://doi.org/10.1089/jpm.2006.0148

Casas, J., Jeppesen, A., Peters, L., Schuelke, T., Magdoza, N. R. K., Hesselgrave, J., & Loftis, L. (2021). Using Quality Improvement Science to Create a Navigator in the Electronic Health Record for the Consolidation of Patient

Information Surrounding Pediatric End-of-Life Care. *Journal of Pain and Symptom Management*, *62*(3), E218–E224. https://doi.org/10.1016/j.jpainsymman.2021.04.006

Clarke, P., & Booth, D. (2011). Copying medical summaries on deceased infants to bereaved parents. *Acta Paediatrica, International Journal of Paediatrics*, *100*(9), 1262–1266. https://doi.org/10.1111/j.1651-2227.2011.02276.x

Cremer, R., Binoche, A., Noizet, O., Fourier, C., Leteurtre, S., Moutel, G., & Leclerc, F. (2007). Are the GFRUP's recommendations for withholding or withdrawing treatments in critically ill children applicable? Results of a two-year survey. *Journal of Medical Ethics*, *33*(3), 128–133. https://doi.org/10.1136/jme.2006.015990

Czynski, A. J., Souza, M., & Lechner, B. E. (2022). The Mother Baby Comfort Care Pathway: The Development of a Rooming-In-Based Perinatal Palliative Care Program. *Advances in Neonatal Care : Official Journal of the National Association of Neonatal Nurses*, *22*(2), 119–124. https://doi.org/10.1097/ANC.0000000000000838

Delgado-Corcoran, C., Wawrzynski, S. E., Mansfield, K., Fuchs, E., Yeates, C., Flaherty, B. F., Harousseau, M., Cook, L., & Epps, J. V. (2023). Grieving Children' Death in an Intensive Care Unit: Implementation of a Standardized Process. *Journal of Palliative Medicine*, *27*(2), 236-NA. https://doi.org/10.1089/jpm.2023.0134

Drolet, C., Roy, H., Laflamme, J., & Marcotte, M.-E. (2016). Feasibility of a Comfort Care Protocol Using Oral Transmucosal Medication Delivery in a Palliative Neonatal Population. *Journal of Palliative Medicine*, *19*(4), 442–450. https://doi.org/10.1089/jpm.2015.0045

Harmon, R. J., Glicken, A. D., & Siegel, R. R. (1984). Neonatal loss in the intensive care nursery. Effects of maternal grieving and a program for intervention. *Journal of the American Academy of Child Psychiatry*, *23*(1), 68–71.

Harris, L. L., Placencia, F. X., Arnold, J. L., Minard, C. G., Harris, T. B., & Haidet, P. M. (2015). A Structured End-of-Life Curriculum for Neonatal-Perinatal Postdoctoral Fellows. *The American Journal of Hospice & Palliative Care*, *32*(3), 253–261. https://doi.org/10.1177/1049909114523825

Haut, C. M., Michael, M., & Moloney-Harmon, P. (2012). Implementing a Program to Improve Pediatric and Pediatric ICU Nurses' Knowledge of and Attitudes Toward Palliative Care. *Journal of Hospice & Palliative Nursing*, *14*(1), 71–79. https://doi.org/10.1097/NJH.0b013e318236df44

Hawes, K., Goldstein, J., Vessella, S., Tucker, R., & Lechner, B. E. (2022). Providing Support for Neonatal Intensive Care Unit Health Care Professionals: A Bereavement Debriefing Program. *American Journal of Perinatology*, *39*(4), 401–408. https://doi.org/10.1055/s-0040-1716481

Ishak Tayoob, M., Rayala, S., Doherty, M., Singh, H. B., Alimelu, M., Lingaldinna, S., & Palat, G. (2024). Palliative Care for Newborns in India: Patterns of Care in a Neonatal Palliative Care Program at a Tertiary Government Children's Hospital. *Health Services Insights*, *17*(NA), 11786329231222858-NA. https://doi.org/10.1177/11786329231222858

Johnson, E. M., Hamilton, M. F., Watson, R. S., Claxton, R., Barnett, M., Thompson, A. E., & Arnold, R. (2017). An intensive, simulation-based communication course for pediatric critical care medicine fellows. *Pediatric Critical Care Medicine*, *18*(8), e348–e355. https://doi.org/10.1097/PCC.0000000000001241

Kang, H. J., & Bang, K. S. (2017). Development and Evaluation of a Self-Reflection Program for Intensive Care Unit Nurses Who Have Experienced the Death of Pediatric Patients. *Journal of Korean Academy of Nursing*, *47*(3), 392–405. https://doi.org/10.4040/jkan.2017.47.3.392

Knighting, K., Kirton, J., Silverio, S. A., & Shaw, B. N. J. (2019). A Network Approach to Neonatal Palliative Care Education Impact on Knowledge, Efficacy, and Clinical Practice. *Journal of Perinatal & Neonatal Nursing*, *33*(4), 350–360. https://doi.org/10.1097/jpn.0000000000000437

Kwon, J. E., & Kim, Y. H. (2023). Changes in the End-of-Life Process in Patients with Life-Limiting Diseases through the Intervention of the Pediatric Palliative Care Team. *Journal of Clinical Medicine*, *12*(20), 6588. https://doi.org/10.3390/jcm12206588

Kymre, I. G., & Bondas, T. (2013). Skin-to-skin care for dying preterm newborns and their parents—A phenomenological study from the perspective of NICU nurses. *Scandinavian Journal of Caring Sciences*, *27*(3), 669–676. https://doi.org/10.1111/j.1471-6712.2012.01076.x

Martel, S. L., & Ives-Baine, L. (2014). “Most Prized Possessions”: Photography as Living Relationships Within the End-of-Life Care of Newborns. *Illness, Crisis & Loss*, *22*(4), 311–332. https://doi.org/10.2190/IL.22.4.d

Meert, K. L., Eggly, S., Kavanaugh, K., Berg, R. A., Wessel, D. L., Newth, C. J. L., Shanley, T. P., Harrison, R., Dalton, H., Michael Dean, J., Doctor, A., Jenkins, T., Park, C. L., Reardon, J., Tomanio, E., Bel, A. L., Villa, M., Pawluszka, A., DiLiberto, M. A., … Burr, J. (2015). Meaning making during parent-physician bereavement meetings after a child’s death. *Health Psychology*, *34*(4), 453–461. https://doi.org/10.1037/hea0000153

Morillo Palomo, A., Clotet Caba, J., Camprubi Camprubi, M., Blanco Diez, E., Silla Gil, J., & Riverola de Veciana, A. (2023). Implementing Palliative care, based on family-centered care, in a highly complex neonatal unit. *Jornal de Pediatria*. https://doi.org/10.1016/j.jped.2023.09.009

Murakami, M., Yokoo, K., Ozawa, M., Fujimoto, S., Funaba, Y., & Hattori, M. (2015). Development of a Neonatal End-of-Life Care Education Program for NICU Nurses in Japan. *Journal of Obstetric, Gynecologic, and Neonatal Nursing : JOGNN / NAACOG*, *44*(4), 481–491. https://doi.org/10.1111/1552-6909.12569

Nellis, M. E., Howell, J. D., Ching, K., & Bylund, C. (2017). The Use of Simulation to Improve Resident Communication and Personal Experience at End-of-Life Care. *Journal of Pediatric Intensive Care*, *6*(2), 91–97. https://doi.org/10.1055/s-0036-1584684

Nesbit, M. J., Hill, M., & Peterson, N. (1997). A comprehensive pediatric bereavement program: The patterns of your life. *Critical Care Nursing Quarterly*, *20*(2), 48–62. https://doi.org/10.1097/00002727-199708000-00010

Parry, S. M., Staenberg, B., & Weaver, M. S. (2018). Mindful Movement: Tai Chi, Gentle Yoga, and Qi Gong for Hospitalized Pediatric Palliative Care Patients and Family Members. *Journal of Palliative Medicine*, *21*(9), 1212–1213. https://doi.org/10.1089/jpm.2018.0203

Rogers, S., Babgi, A., & Gomez, C. (2008). Educational interventions in end-of-life care: Part I: An educational intervention responding to the moral distress of NICU nurses provided by an ethics consultation team. *Advances in Neonatal Care*, *8*(1), 56–65. https://doi.org/10.1097/01.ANC.0000311017.02005.20

Rushton, C. H., Reder, E., Hall, B., Comello, K., Sellers, D. E., & Hutton, N. (2006). Interdisciplinary interventions to improve pediatric palliative care and reduce health care professional suffering. *Journal of Palliative Medicine*, *9*(4), 922–933. https://doi.org/10.1089/jpm.2006.9.922

Sabaq, A. G., & Khalaf, S. M. (2016). Effect of Educational Program on Nurses' Performance Regarding Neonatal Palliative Care. *NA*, *10*(1), 43–71.

Samsel, C., & Lechner, B. E. (2015). End-of-life care in a regional level IV neonatal intensive care unit after implementation of a palliative care initiative. *Journal of Perinatology*, *35*(3), 223–228. https://doi.org/10.1038/jp.2014.189

Scheurer, J. M., Norbie, E., Bye, J. K., Villacis-Calderon, D., Heith, C., Woll, A., Shu, D., McManimon, K., Kamrath, H., & Goloff, N. (2023). Pediatric End-of-Life Care Skills Workshop: A Novel, Deliberate Practice Approach. *Academic Pediatrics*, *23*(5), 860–865. https://doi.org/10.1016/j.acap.2022.11.006

Tripathi, S., Laksana, E., McCrory, M. C., Hsu, S., Zhou, A. X., Burkiewicz, K., Ledbetter, D. R., Aczon, M. D., Shah, S., Siegel, L., Fainberg, N., Morrow, K. R., Avesar, M., Chandnani, H. K., Shah, J., Pringle, C., & Winter, M. C. (2023). Analgesia and Sedation at Terminal Extubation: A Secondary Analysis from Death One Hour after Terminal Extubation Study Data*. *Pediatric Critical Care Medicine*, *24*(6), 463–472. https://doi.org/10.1097/PCC.0000000000003209

Twamley, K., Kelly, P., Moss, R., Mancini, A., Craig, F., Koh, M., Polonsky, R., & Bluebond-Langner, M. (2013). Palliative care education in neonatal units: Impact on knowledge and attitudes. *BMJ Supportive & Palliative Care*, *3*(2), 213–220. https://doi.org/10.1136/bmjspcare-2012-000336

Vesely, C., Newman, V., Winters, Y., & Flori, H. (2017). Bringing Home to the Hospital: Development of the Reflection Room and Provider Perspectives. *Journal of Palliative Medicine*, *20*(2), 120–126. https://doi.org/10.1089/jpm.2016.0070

Williams, B. K., Pendergrass, T. L., Grooms, T. R., & Florez, A. R. (2021). End of Life Simulation in a Pediatric Cardiac Intensive Care Unit. *Clinical Simulation in Nursing*, *60*, 3–10. https://doi.org/10.1016/j.ecns.2021.06.007

Wolff, T., Dorsett, C., Connolly, A., Kelly, N., Turnbull, J., Deorukhkar, A., Clements, H., Griffin, H., Chhaochharia, A., Haynes, S., Webb, K., & Manning, J. C. (2021). Chameleon project: A children's end-of-life care quality improvement project. *Bmj Open Quality*, *10*(4), 9. https://doi.org/10.1136/bmjoq-2021-001520

Woolgar, F., & Archibald, S.-J. (2021). An exploration of Neonatal Intensive Care Unit (NICU) staff experiences of attending pre-brief and debrief groups surrounding a patient's death or redirection of care. *Journal of Neonatal Nursing*, *27*(5), 352–357. https://doi.org/10.1016/j.jnn.2021.03.007

Younge, N., Smith, P. B., Goldberg, R. N., Brandon, D. H., Simmons, C., Cotten, C. M., & Bidegain, M. (2015). Impact of a palliative care program on end-of-life care in a neonatal intensive care unit. *Journal of Perinatology*, *35*(3), 218–222. https://doi.org/10.1038/jp.2014.193

Zhang, R., Tang, Q., Zhu, L. H., Peng, X. M., Zhang, N., Xiong, Y. E., Chen, M. H., Chen, K. L., Luo, D., Li, X., & Latour, J. M. (2022). Testing a Family Supportive End of Life Care Intervention in a Chinese Neonatal Intensive Care Unit: A Quasi-experimental Study With a Non-randomized Controlled Trial Design. *Frontiers in Pediatrics*, *10*, 870382. https://doi.org/10.3389/fped.2022.870382

Mixed Methods Appraisal Tool Results

Title	Authors:	Study design	Are there clear research questions?	Do the collected data allow to address the research questions?	Are the participants representative of the target population?	Are measurements appropriate ?	Are there complete outcome data?	Are the confounders accounted for in the design and analysis?	Is the intervention administered (or exposure occurred) as intended?
Quantitative non-randomized									
Implementing Palliative care, based on family-centered care, in a highly complex neonatal unit	Morillo Palomo, et al	quantitative non-randomized	Yes	Yes	yes	yes	yes	yes	yes
Development of a Neonatal End-of-Life Care Education Program for NICU Nurses in Japan	Murakami, et al	quantitative non-randomized	Yes	Yes	can't tell	yes	yes	no	yes
The Use of Simulation to Improve Resident Communication and Personal Experience at End-of-Life Care	Nellis, et al	quantitative non-randomized	Yes	Yes	can't tell	yes	no	no	yes
Mindful Movement: Tai Chi, Gentle Yoga, and Qi Gong for Hospitalized Pediatric Palliative Care Patients and Family Members	Parry,et al	quantitative non-randomized	Yes	Yes	can't tell	yes	yes	no	yes
Educational interventions in end-of-life care: Part I: An educational intervention responding to the moral distress of NICU nurses provided by an ethics consultation team	Rogers, et al	quantitative non-randomized	Yes	Yes	can't tell	yes	yes	can't tell	yes
Pediatric End-of-Life Care Skills Workshop: A Novel, Deliberate Practice Approach	Scheurer, et al	quantitative non-randomized	Yes	Yes	can't tell	yes	yes	no	yes
Palliative care education in neonatal units: impact on knowledge and attitudes	Twamley, et al	quantitative non-randomized	Yes	Yes	can't tell	yes	no	no	yes
Testing a Family Supportive End of Life Care Intervention in a Chinese Neonatal Intensive Care Unit: A Quasi-experimental Study With a Non-randomized Controlled Trial Design	Zhang, R. et al	quantitative non-randomized	Yes	Yes	yes	yes	yes	no	yes
Impact of educational programs on nurses' knowledge and attitude toward pediatric palliative care	Abuhammad, et al	quantitative non-randomized	Yes	Yes	can't tell	yes	yes	no	yes
Improving Neonatal Intensive Care Unit Providers' Perceptions of Palliative Care through a Weekly Case-Based Discussion	Allen, et al	quantitative non-randomized	Yes	Yes	yes	yes	no	no	yes
A Curriculum to Improve Residents' End-of-Life Communication and Pain Management Skills During Pediatrics Intensive Care Rotation: Pilot Study	Asuncion, Arsenia et al	quantitative non-randomized	Yes	Yes	can't tell	yes	yes	can't tell	yes
The Use of Dexmedetomidine in Pediatric Palliative Care: A Preliminary Study	Burns, et al	quantitative non-randomized	Yes	Yes	yes	yes	yes	no	yes
A Structured End-of-Life Curriculum for Neonatal-Perinatal Postdoctoral Fellows	Harris, et al	quantitative non-randomized	Yes	Yes	can't tell	yes	yes	no	yes
Implementing a Program to Improve Pediatric and Pediatric ICU Nurses' Knowledge of and Attitudes Toward Palliative Care	Haut, et al	quantitative non-randomized	Yes	Yes	can't tell	yes	yes	no	yes
Providing Support for Neonatal Intensive Care Unit Health Care Professionals: A Bereavement Debriefing Program	Hawes, et al	quantitative non-randomized	Yes	Yes	can't tell	yes	yes	no	yes
Changes in the End-of-Life Process in Patients with Life-Limiting Diseases through the Intervention of the Pediatric Palliative Care Team	Kwon, et al	quantitative non-randomized	Yes	Can't tell	yes	can't tell	no	no	can't tell
Effect of Educational Program on Nurses' Performance Regarding Neonatal Palliative Care	Sabaq, et al	quantitative non-randomized	Yes	Yes	can't tell	yes	yes	no	yes

Title	Authors:	Study design	Are there clear research questions?	Do the collected data allow to address the research questions?	Is the sampling strategy relevant to address the research question?	Is the sample representative of the target population?	Are the measurements appropriate?	Is the risk of nonresponse bias low?	Is the statistical analysis appropriate to answer the research question?
**Quantitative descriptive**									
End-of-life care in a regional level IV neonatal intensive care unit after implementation of a palliative care initiative	Samsel, et al	quantitative descriptive	Yes	Yes	yes	yes	yes	yes	yes
Analgesia and Sedation at Terminal Extubation: A Secondary Analysis from Death One Hour after Terminal Extubation Study Data*	Tripathi, et al	quantitative descriptive	Yes	Yes	yes	can't tell	yes	yes	yes
Bringing Home to the Hospital: Development of the Reflection Room and Provider Perspectives	Vesely,et al.	quantitative descriptive	Yes	Yes	yes	can't tell	yes	no	yes
Impact of a palliative care program on end-of-life care in a neonatal intensive care unit	Younge, et al	quantitative descriptive	Yes	Yes	yes	yes	yes	yes	yes
The wrap-up: A unique forum to support pediatric residents when faced with the death of a child	Bateman, et al.	quantitative descriptive	Yes	Yes	yes	can't tell	yes	can't tell	yes
Utility of morbidity and mortality conference in end-of-life education in the neonatal intensive care unit	Carter, et al	quantitative descriptive	Yes	Yes	can't tell	can't tell	yes	yes	yes
Copying medical summaries on deceased infants to bereaved parents	Clarke, et al	quantitative descriptive	Yes	Yes	yes	can't tell	yes	can't tell	yes
Are the GFRUP's recommendations for withholding or withdrawing treatments in critically ill children applicable? Results of a two-year survey	Cremer,et al	quantitative descriptive	Yes		yes	yes	yes	yes	yes
The Mother Baby Comfort Care Pathway: The Development of a Rooming-In-Based Perinatal Palliative Care Program	Czynski, et al	quantitative descriptive	can't tell	Can't tell	can't tell	yes	can't tell	can't tell	yes
Feasibility of a Comfort Care Protocol Using Oral Transmucosal Medication Delivery in a Palliative Neonatal Population	Drolet, et al	quantitative descriptive	Yes	Yes	yes	yes	yes	yes	yes
An intensive, simulation-based communication course for pediatric critical care medicine fellows	Johnson, et al	quantitative descriptive	Yes	Yes	yes	no	yes	yes	yes
Simulation-Based Palliative Care Communication for Pediatric Critical Care Fellows:	Brock, et al	quantitative descriptive	No	No	yes	yes	can't tell	can't tell	can't tell
Grieving Children' Death in an Intensive Care Unit: Implementation of a Standardized Process.	Delgado-Corcoran, et al	quantitative descriptive	Yes	Yes	yes	can't tell	yes	no	yes

Title	Authors:	Study design	Are there clear research questions?	Do the collected data allow to address the research questions?	Is the qualitative approach appropriate to answer the research question?	Are the qualitative data collection methods adequate to address the research question?	Are the findings adequately derived from the data?	Is the interpretation of results sufficiently substantiated by data?	Is there coherence between qualitative data sources, collection, analysis and interpretation?
Qualitative									
"Most Prized Possessions": Photography as Living Relationships Within the End-of-Life Care of Newborns	Martel, et al	qualitative	Yes	Yes	yes	yes	yes	yes	yes
Meaning making during parent-physician bereavement meetings after a child's death	Meert, et al	qualitative	Yes	Yes	yes	yes	yes	yes	yes
Bereaved mothers' and fathers' perceptions of a legacy intervention for parents of infants in the NICU	Akard,et al	qualitative	Yes	Yes	yes	yes	yes	no	yes
Examining Palliative Care Team Involvement in Automatic Consultations for Children on Extracorporeal Life Support in the Pediatric Intensive Care Unit	Doorenbos, et al	qualitative	Yes	Yes	yes	yes	yes	yes	yes
Neonatal loss in the intensive care nursery. Effects of maternal grieving and a program for intervention	Harmon, et al	qualitative	No	Can't tell	can't tell	yes	can't tell	can't tell	can't tell
Skin-to-skin care for dying preterm newborns and their parents - A phenomenological study from the perspective of NICU nurses	Kymre,et al	qualitative	Yes	Yes	yes	yes	yes	yes	yes
**Title**	**Authors:**	**Study design**	**Are there clear research questions?**	**Do the collected data allow to address the research questions?**	**Is there an adequate rationale for using a mixed methods design?**	**Are the different components of the study effectively integrated?**	**Are outputs of the integration adequately interpreted?**	**Are inconsistencies between quantitative and qualitative results adequately addressed?**	**Do the different components of the study adhere to the quality criteria of each tradition of the methods involved?**
Mixed Methods									
Development and Evaluation of a Self-Reflection Program for Intensive Care Unit Nurses Who Have Experienced the Death of Pediatric Patients	Kang, et al	mixed methods	Yes	Yes	yes	yes	yes	can't tell	can't tell
A Network Approach to Neonatal Palliative Care Education Impact on Knowledge, Efficacy, and Clinical Practice	Knighting, et al	mixed methods	Yes	Yes	yes	no	no	no	yes
An exploration of Neonatal Intensive Care Unit (NICU) staff experiences of attending pre-brief and debrief groups surrounding a patient's death or redirection of care	Woolgar, et al	mixed methods	Yes	Yes	no	no	no	no	yes
